# A review and perspective on the assessment, management and prevention of fragility fractures in patients with osteoporosis and chronic kidney disease

**DOI:** 10.1007/s12020-021-02735-9

**Published:** 2021-05-11

**Authors:** Geeta Hampson, Grahame J. Elder, Martine Cohen-Solal, Bo Abrahamsen

**Affiliations:** 1 Department of Chemical Pathology and Metabolic Medicine, St Thomas’ Hospital, London, UK; 2 Metabolic Bone Clinic, Department of Rheumatology, Guy’s Hospital, London, UK; 3 Department of Renal Medicine, Westmead Hospital, Sydney, New South Wales Australia; 4 Osteoporosis and Bone Biology Program, Garvan Institute of Medical Research, Sydney, New South Wales Australia; 5 Faculty of Medicine, University of Notre Dame Australia, Level 2, 88-90 Water Street, Auburn, New South Wales 2144 Australia; 6 Bioscar Inserm U1132 and Université de Paris, Hôpital Lariboisière, F-75010 Paris, France; 7 Department of Medicine, Holbæk Hospital, Holbæk, Denmark; 8 Department of Clinical Research, Open Data Explorative Network, University of Southern Denmark, Odense, Denmark

**Keywords:** Chronic kidney disease, Dialysis, Fracture risk, Osteoporosis drugs

## Abstract

This article aims to review the methods used for the assessment of fracture risk and the use of osteoporosis medications for fracture prevention in the population with CKD, and highlights the difficulties faced by clinicians in the management of these patients and the latest recommendations and guidelines. Chronic kidney disease (CKD) and osteoporosis often co-exist in older adults, and they present a major healthcare challenge. CKD mineral and bone disorder (CKD-MBD) occurs as renal function declines and this syndrome affects most patients in CKD stages 4 and 5. The biochemical abnormalities of CKD-MBD, renal bone disease and risk factors associated with age-related bone loss and osteoporosis lead to a cumulative effect on fracture risk and mortality. There is a need for routine evaluation of fracture risk and fracture prevention in this population. Measurement of bone mineral density (BMD) and the use of the FRAX tool have predictive value for incident fractures in the general population and in CKD. This enables physicians to identify CKD patients most at risk of sustaining a fragility fracture and allows a more targeted approach to fracture prevention. Data analysis from the pivotal trials of therapeutic agents used in osteoporosis show that these drugs can be considered in mild and moderate CKD (stages 1–3 CKD). Off-label drug use in patients with CKD-MBD and more severe renal impairment (CKD stages 4 and 5) could offer significant benefits to sub-groups of patients when carefully tailored to each individual’s bone turnover and calcium and phosphate balance. However, this requires a selective approach and treatment decisions based on inference from pathophysiology while we await further trials. Guidelines advocate the correction and/or reduction of the biochemical abnormalities of CKD-MBD before initiation of treatment with osteoporosis drugs and close monitoring during treatment.

## Introduction

Osteoporosis is a progressive systemic skeletal disorder characterised by low bone mass or bone mineral density (BMD) and microarchitectural deterioration, which leads to increased skeletal fragility. The World Health Organisation defines osteoporosis as BMD, measured by dual-energy X-ray absorptiometry (DXA), that is 2.5 standard deviations (SDs) or more below the young adult mean value for women (*T* score equal to or less than –2.5). Population studies show that the risk of fracture increases with decreasing BMD with an approximately twofold increase in fracture risk for each SD decrease in BMD [1].

Chronic kidney disease (CKD) leads to significant derangements in bone metabolism termed renal osteodystrophy (ROD). Based on bone biopsy abnormalities, these may include high turnover (hyperparathyroid bone disease), low bone turnover or adynamic bone disease (ABD), osteomalacia and mixed uraemic osteodystrophy. ROD is now a component of the CKD mineral and bone disorder (CKD-MBD) syndrome, which groups disturbances of calcium, phosphate, parathyroid hormone (PTH) and vitamin D metabolism, cardiovascular calcification and bone abnormalities and results in an increased risk of cardiovascular events, fracture and mortality [2]. The 2017 KDIGO guidelines and the 2020 Consensus Statement from the International Osteoporosis Foundation (IOF) and European Dialysis and Transplant Association (ERA-EDTA) integrate skeletal events such as fractures, and evaluation of fracture risk, as targets in the management of ROD [3, 4]. The metabolic effects of CKD can challenge the mechanical competence of an otherwise healthy skeleton, but as most patients with moderate or severe CKD are elderly, the bone status of patients is often already affected by age-related or post-menopausal osteoporosis. Critically, the risk of fractures in CKD combine classic osteoporosis risk factors of advancing age, low body mass index (BMI) and prior fracture with secondary risk factors such as glucocorticoid use and inflammatory diseases, plus metabolic derangements associated specifically with CKD-MBD and the therapeutics used in moderate to severe CKD management. All these have the potential to affect bone strength and many also affect the risk of falls. These combinations of predisposing factors may have a cumulative effect on fracture risk in people with CKD.

Assessment of fracture risk and treatment for fracture prevention in CKD stages 4 and 5 is particularly complex and challenging. A point very well made in the IOF/ERA-EDTA joint consensus statement is that action is essential to move beyond current variations in care and treatment nihilism [4]. The aim of this article is to review methodologies for the assessment of fracture risk, and the use of osteoporosis medications for fracture prevention in CKD including where a case can be made for off-label prescribing.

## Epidemiology

Both osteoporosis and CKD are common conditions, which often co-exist and have an increasing global prevalence. Osteoporosis is the most widespread bone disease, affecting one in two post-menopausal women and one in five middle-aged men. Reports from the IOF on the economic burden of osteoporosis show that the cost of osteoporosis is 37 billion € per year in the EU, and US $19 billion per year in the USA [5]. Costs are projected to rise dramatically alongside an increasing osteoporosis prevalence in coming years, with an increased proportion of the population of both industrialised and less developed nations becoming elderly (>65 years). In terms of morbidity, osteoporotic fractures cause an annual global loss of 5.8 million healthy life years due to disability and reduced relative survival. Hip fractures are associated with a 30% mortality at 1 year and 53% of patients who sustain a hip fracture are no longer able to live independently [6].

CKD increases in prevalence with ageing, is more common amongst women and has an estimated global prevalence of 11–13% [7]. However, males are more likely to receive dialysis and kidney transplantation [8]. Bone management in the presence of reduced renal function is a common challenge in osteoporosis and fracture liaison service clinics. This is unsurprising, as NHANES III indicates that more than 60% of women with a diagnosis of osteoporosis had CKD stage 3 (estimated glomerular filtration rate (eGFR) 30–59 mL/min) and 23% had CKD stage 4 (eGFR 15–29 mL/min). The proportion of patients aged between 70 and 79 years who have osteoporosis and an eGFR < 35 mL/min is 21%, rising to 54% in the over 80s. However, a reduced eGFR may constitute ‘normal ageing’ rather than specific pathology in a proportion of this population [9]. Several large cohort studies show that fracture incidence increases progressively by CKD stages [10, 11] and lower eGFR is associated with a higher risk of both hip fracture and non-vertebral fractures [12, 13]. Excess fracture risk is also seen following kidney transplantation. Data from NHANES indicate that ~24% of CKD is related to DM [14], and DM increases fracture risk independently of renal function [15]. Hence, fracture risk may be even higher in those with CKD and DM. A Danish national register-based study found that patients on dialysis experienced more fractures than renal transplant patients, but when adjusted for age, sex, prior fractures and comorbid conditions, both groups had a similarly increased risk, at just under twofold that of the background population [16]. In a study from North East Scotland, CKD stages 3–5 was associated with an increased incidence of hip fracture admissions, with rates of 10.0 (95% CI 9.4–10.7) per 1000 patient-years compared to 1.5 (95% CI 1.2–1.7) in those with normal eGFR [17]. A further study in dialysis patients, the Dialysis Outcomes and Practice Patterns Study (DOPPS), showed an increase in fractures, including hip fractures, ranging from 1.5-fold to 8-fold that of the general population across all countries [18]. Just as in the general population, vertebral fractures are under-reported in CKD and dialysis cohorts, because vertebral imaging is performed relatively infrequently, and even actively discouraged in family medicine [19]. Hence, vertebral fractures may not be recognised as fracture events in CKD and dialysis patients, despite being associated with poorer clinical outcomes. Mortality rates post-hip fracture are higher in CKD patients in hospital and at 1 year compared to those with normal renal function. A 3.7-fold increase in the unadjusted relative risk of death and a 4-fold increase in death/rehospitalisation and longer hospital stay have been observed in dialysis patients who have a fracture compared with those with no fractures [20].

This poses a significant health challenge, and is a burden on health and social care resources. There is thus a need for the implementation of better fracture prevention strategies in this high-risk population.

## Assessment of bone health in CKD and ESRD

### Bone biopsy

Although the latest KDIGO guidelines no longer recommend bone biopsy as mandatory before starting anti-resorptive treatment in patients with CKD, bone histomorphometry remains the ‘gold standard’ for the assessment of abnormalities of bone turnover, mineralisation and volume (TMV) associated with CKD-MBD. Bone biopsy using a trephine with an internal diameter of 7–8 mm has become infrequent, because patients are reluctant to undergo this relatively invasive procedure, few centres offer bone biopsy and histomorphometric analyses [21] and slow processing may lead to a delayed diagnosis. In addition, current recommendations only suggest bone biopsy for patients with CKD-MBD if the result will influence treatment decisions. However, in recent years the procedure has become simpler, and use of a smaller diameter biopsy needle rather than the classic Bordier or Rochester trephine is absolutely feasible [21]. Histology can differentiate between high turnover (generally due increased PTH) and low bone turnover states, both of which have been linked to adverse skeletal outcomes [2] and is the only method that can show alterations of mineralisation. A recent study showed that measuring half of a full 7.5 mm biopsy yielded an accurate diagnosis of ROD, in a comparable manner to the full core biopsy. Good correlations were found for static and dynamic parameters, although exact comparison to the smaller Jamshidi bone biopsy sample was not fully addressed [22].

There is no recent update on the prevalence of high versus low turnover bone disease in CKD, and there have been conflicting data due, in part, to recruitment bias and the conduct of biopsies in symptomatic patients for clinical rather than epidemiological purposes [15]. A decade ago, histological features of high bone turnover were described in about half of patients with CKD stages 3 and 4 and in 61% with CKD stage 5 [23]. By contrast, concurrent small studies reported a relatively high prevalence of low turnover bone diseases in patients who were pre-dialysis, with a prevalence up to 88% in CKD stage 3 and 78% in CKD stage 4 [24]. In a large study of 630 bone biopsies from adult patients on dialysis published in 2011, 58% showed low bone turnover, 18% had normal turnover and 24% exhibited high bone turnover [25].

Importantly, the histological characteristics of bone from CKD patients who fracture and whether a bone biopsy diagnosis will improve management to prevent clinical fractures, hospitalisation and mortality are unknown. A recent study examined this issue in 260 patients with CKD stages 3–5D with 12–30 months follow up [26]. Bone histology was available in 67% of patients. The authors found that osteitis fibrosa was the most prevalent form and present in 51% of patients. There was a significant association between bone pain in patients with low trabecular bone volume, but no other significant association between the histological diagnoses based on the turnover, TMV [25] and clinical symptoms, or outcomes of fracture, hospitalisation and mortality. However, the number of fractures in the study population during follow up was too low (*n* = 7) to draw conclusions regarding classification of ROD and fracture risk. One study that based bisphosphonate, cinacalcet or teriparatide treatment on bone biopsy results reported improved BMD in patients treated with teriparatide, but not in the other groups, after an average treatment period of 13–16 months [27]. Thus, we have no current evidence that bone biopsy findings predict outcomes, the relationship between the types of ROD and fractures remain uncertain [28]. It can be argued that the variables derived from histomorphometry such as bone formation or mineralisation rates may not be the prime determining factors of fracture risk in CKD. On the other hand, bone biopsy might assist in avoiding the inappropriate use of anti-resorptive agents in patients with low bone turnover or osteomalacia, for whom other treatments should be considered. Although the use of bone biopsies is still supported in advanced CKD, it is important to ascertain whether alternative, non-invasive diagnostic tools and biomarkers may be useful surrogates in fracture prediction.

### BMD by DXA

In the general population, measurement of BMD by DXA is used routinely in the diagnosis of osteoporosis and to assess fracture risk, and inclusion of BMD, together with clinical risk factors in the fracture risk assessment tool (FRAX) algorithm, improves its predictive value [29]. However, in patients with CKD, the use of BMD in fracture prediction has been controversial, as it has several limitations. It does not capture measures of bone quality such as bone micro-architecture, and does not differentiate between trabecular and cortical bone, the latter being more significantly affected in CKD [30]. Another limitation is overestimation at the lumbar spine in cases of osteoarthritis, spine deformity and vascular calcification [31]. Despite its limitations, measurement of BMD to assess fracture risk has been recommended in the revised 2017 KDIGO guidelines in patients with CKD stages 3–5D with evidence of CKD-MBD and/or in the presence of risk factors for osteoporosis [3]. This recommendation is based on data from a meta-analysis of 13 studies and 4 prospective cohort studies, which showed that BMD was significantly lower in patients with fractures, and can predict fracture risk in CKD stages 3–5D [32, 33].

Other data can be acquired from DXA scan images of the lumbar spine. The trabecular bone score (TBS) provides an indirect measure of bone structure. TBS has not been widely used in the context of CKD, although a prospective cohort study; the Canadian Multicentre Osteoporosis Study (CaMos), showed that the TBS was associated with increased fracture risk independent of BMD in patients with reduced GFR (<60 ml/min) [34]. However, only 2.5% of the study population had CKD stage 4 and none had CKD stages 5–5D. In dialysis patients, a lower TBS has been associated with higher bone turnover and prevalent fracture [35]. In a recent prospective study in patients on haemodialysis, fracture incidence, death and cardiovascular events were higher in those with a lower TBS [36].

Hip structural analysis (HSA) using the DXA analysis software has been shown to be useful in the evaluation of hip geometry, hip axis length (HAL) and mechanical strength, and studies have indicated that these parameters are good predictors of hip fracture [37]. Using HSA, cortical thickness at the femoral neck and shaft were found to be reduced. The buckling ratio (derived as femoral neck radius/cortical thickness) was increased in ESRD compared to age and sex-matched controls, suggestive of changes to hip geometry related to altered bone remodelling [38]. Further studies are needed to assess whether CKD-related changes to hip geometry can be used in fracture prediction and treatment decisions.

### High-resolution peripheral quantitative computed tomography (HR-pQCT) measurement

HR-pQCT allows the discrimination of trabecular and cortical bone and may prove useful in fracture risk assessment in CKD where abnormalities in cortical bone structure such as cortical thinning and porosity are present and contribute to bone fragility [39, 40]. A longitudinal study of patients with CKD, using HR-pQCT, showed significant reductions in cortical area, density and thickness at the distal radius, and increases in cortical porosity and trabecular area associated with hyperparathyroidism [41]. A relationship between HR-pQCT and bone biopsy has been reported in dialysis patients, particularly in the cortical compartment. The study also showed correlations between HR-pQCT trabecular density, bone volume/tissue volume, trabecular number, separation and thickness at the distal radius and bone biopsy. Increased cortical porosity on bone histomorphometry was associated with lower cortical density at the distal radius [42]. In another study of 43 patients with CKD 4–5, negative correlations were observed between distal radius HR-pQCT parameters and bone turnover from histomorphometry [43]. Although preliminary, these data suggest that in advanced CKD, imaging cortical sites using HR-pQCT may provide useful information for the assessment of fracture risk, although the technique is not widely available and used mostly for research.

### Laboratory investigations

#### Parathyroid hormone

**Fig. 1:**
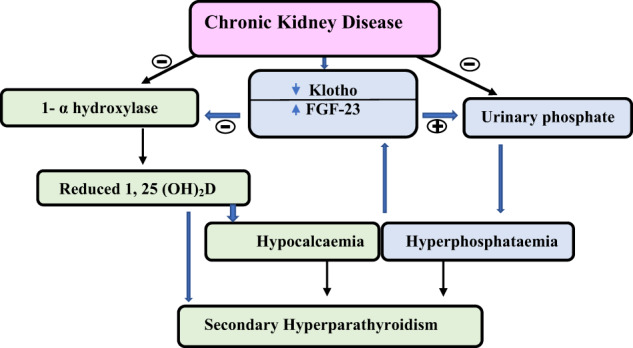
Pathogenesis of secondary hyperparathyroidism in chronic kidney disease (CKD)

Serum PTH is central to the pathogenesis of renal bone disease [44, 45] and values generally increase when eGFR falls below 60 mL/min/1.73 m^2^. This initially homoeostatic secondary hyperparathyroidism prevents hypocalcaemia and stimulates renal phosphate excretion. However, with a progressive decline in GFR, serum phosphate starts to rise, contributing to a further hypocalcaemic drive, increased production of fibroblast growth factor-23 (FGF-23) and further increases in PTH. FGF-23 in turn contributes to reduced activity of 1-α-hydroxylase, which leads to decreased 1,25-dihydroxyvitamin D and disinhibits further PTH production, as illustrated in Fig. 1.

PTH is used as a surrogate marker for bone turnover in the absence of bone histomorphometry. Target values for intact PTH (iPTH) have been set for dialysis patients by the National Kidney Foundation/Kidney Disease Outcomes Quality Initiative (NKF/K-DOQI) and KDIGO. PTH concentrations of >300 pg/mL (NKF/K-DOQI) or >9 times the upper limit (UL) of normal (KDIGO) are suggested cut-off points for diagnosing high bone turnover and <150 pg/mL or <2 times the upper limit of normal (ULN) for low bone turnover. These ranges have relatively high specificity but low sensitivity [46]. In a more recent study, the optimal iPTH cut-off value for discriminating high bone turnover was five times the ULN (mean iPTH 347 pg/mL) [43]. Variability in the PTH assays may also account for differing cut-off values between the different guidelines. To derive their diagnostic values, the NKF/K-DOQI used the Nicholls iPTH assay which is no longer in use and has been shown to vary substantially from other commercially available PTH assays [47]. For this reason, the KDIGO guidelines recommend a broader PTH range (2–9-fold) in CKD-5D, although it is important to pay close attention to trends in PTH rather than rely only on absolute values when making clinical decisions.

The optimal PTH concentration remains unclear in patients with CKD 3–5, as some rise in PTH is an appropriate adaptive response to declining kidney function. Thus, the revised KDIGO 2017 guidelines advise against basing clinical decisions on a single PTH measurement. They suggest that if the iPTH is progressively rising or persistently above the normal range, patients should be evaluated for biochemical abnormalities which can be addressed, such as hyperphosphataemia, high dietary phosphate, hypocalcaemia and vitamin D deficiency [3].

In CKD-5D, significantly greater risk of any type of incident fracture has been associated with PTH levels either <150 pg/mL (hazard ratio (HR) = 3.47, *P* < 0.01] or >300 pg/mL (HR = 5.88, *P* < 0.0001) compared with 150–300 pg/mL [48]. There are several reports of an association between PTH level and mortality risk [49–52], although an earlier meta-analysis showed no association [53], and both low and high serum PTH have been associated with higher all cause and cardiovascular mortality in patients on maintenance dialysis. Data from haemodialysis patients after enrolment in DOPPS phases 4 and 5 reported highest mortality associated with PTH values >600 pg/mL, HR, 1.35; 95% confidence interval, 1.20–1.52 versus PTH 200–399 pg/mL [54]. In another retrospective observational cohort study of patients on dialysis, increase in PTH from a baseline value < 150 pg/mL to 150–300 pg/mL was associated with lower mortality, although the study had some limitations due to lack of available data on some measurements which may have led to residual confounding [55]. In conclusion, lower mortality is reported in patients with CKD-5D with PTH between 150 and 300 (2–5 × ULN) [56] or 400 ng/mL (6 × ULN) [46] although these associations do not imply a causal effect. There is an absence of RCT evidence to define optimal PTH levels in CKD stages 3–5, or clinical endpoints of hospitalization, fracture or mortality [3]. However, when PTH values trend towards the range seen in patients on dialysis, many clinicians would certainly consider instituting treatment to reduce further rises.

#### Vitamin D

The serum concentration of 25-hydroxyvitamin D (25(OH)D) is a marker of vitamin D status, although what level of 25(OH)D represents sufficiency is subject to debate. There are no conclusive data as to whether the presence of CKD would alter recommended levels. The 2017 KDIGO guidelines recommend that vitamin D deficiency and insufficiency be corrected in CKD using the same optimal target as for the general population, although optimal targets vary and can range from 20 to 30 ng/mL (50–75 nmol/L) [3, 57]. Some studies show a high prevalence of vitamin D deficiency/insufficiency (<30 ng/L) in CKD, ranging from 40.7% in CKD stage 3, 61.5% in stage 4, and 85.7% in stage 5 [58]. In a cohort study, 79 and 57% out of 908 patients on haemodialyses had 25(OH)D levels of <30 ng/mL (75 nmol/L) and <20 ng/mL (50 nmol/L), respectively [59]. In pre-dialysis CKD, only 29% of patients with CKD stage 3 and 17% of patients with CKD stage 4 were vitamin D sufficient, defined as 25(OH)D > 30 ng/mL (75 nmol/L) [60]. Low 25(OH)D concentrations have been associated with secondary hyperparathyroidism, low BMD and increased bone turnover in CKD and ESRD [61–63]. An increased mortality risk of 30% has been associated with serum 25(OH)D values < 18 ng/mL (45 nmol/L) in dialysis patients, although these effects should take serum PTH and FGF-23 into account, and whether vitamin D has a causative role remains unclear [64]. Higher concentrations of 25(OH)D > 40 ng/mL (100 nmol/L) have been associated with reduced bone turnover with no resultant hypercalcaemia or hyperphosphataemia, but with improvement in muscle strength and falls reduction in CKD stage 5 [65–67]. However, there are sparse data to determine whether there are associations with reduced fractures. In a meta-analysis of patient groups with varying 25(OH)D concentrations, mortality was greater in those with values <10 ng/mL versus >30 ng/mL (<25 nmol/L versus >75 nmol/L), but there is a possibility of publication bias, and low 25(OH)D may be a marker of poor health rather than a causative factor [68]. The effects of vitamin D supplementation in CKD depend on the dose of vitamin D, the dosing protocol, treatment duration, population and baseline 25(OH)D concentration. A meta-analysis of observational studies demonstrated a 41% reduction in PTH in dialysis patients following supplementation, with a mean difference of 25(OH)D of +24 ng/mL (60 nmol/L) [67]. However, these findings were not supported in a more recent meta-analysis where no significant PTH-lowering was seen following vitamin D in pre-dialysis CKD or in ESRD [69]. Nevertheless, administration of native vitamin D in CKD stage 5D may improve bone mineralization [33].

#### Bone biomarkers

Bone turnover markers are either collagen fragments released from bone to the circulation during the process of bone remodelling or enzymes secreted by bone cells active in the remodelling process. Hence, currently available bone formation markers are by-products of collagen synthesis released during the process of bone formation such as procollagen type 1 N-terminal propeptide (PINP) or enzymes produced by osteoblasts; such as bone-specific alkaline phosphatase (bALP) [70]. There are limitations to the use of the collagen pro and telopeptides as they are excreted in urine and therefore are elevated in CKD. This— is particularly true for the resorption marker CTX and to a lesser degree for PINP. BALP is a subset of tissue non-specific ALP, and both are unaffected by renal clearance and reflect bone formation. Total ALP values above 120 IU/L (ULN) are associated with increased mortality in CKD [71]. Total ALP concentration of <88 IU/L (range of total ALP in the control group: 55–78 IU/L) has been shown to discriminate between low versus non-low bone turnover and >102 IU/L between high versus non-high bone turnover in CKD 4–5D, although the positive predictive value was lower than for bALP [43]. Total ALP is widely available and forms part of the liver profile panel and is a cheap enzymatic test. However, total ALP consists of the liver and bone isoenzyme and is raised in cholestatic liver disease, although other liver enzymes such as gamma-glutamyl transferase will also be elevated. Thus, in the context of suspected or co-existing liver disease, measurement of bALP is required to assess the bone contribution to the raised total ALP. BALP has been shown to be superior to total ALP in assessment of bone turnover, although as total ALP is more widely available, it can be used as a screening test, with confirmation using bALP if the results are equivocal and if bALP is available. Note, total ALP can remain within the normal range despite a raised bALP. In previous studies of bone biopsies in CKD stages 4–5D, bALP and PTH were shown to have similar diagnostic ability to predict high bone turnover [43, 46]. PTH and bALP may not be as accurate for diagnosing low bone turnover, but the addition of bALP to PTH improves the diagnostic accuracy for discriminating low from non-low bone turnover [72]. There are several commonly available immunoassays for bALP which report results either as mass units (µg/L) or activity (U/L). Reference ranges vary depending on the assay used. In studies using assays which report activity units, the reference range was 15.0–41.3 U/L for men, 14.2–42.7 U/L for post-menopausal women and 11.6–29.6 U/L for premenopausal women. The authors found that bALP levels < 33.1 U/L discriminated low from non-low bone turnover and >42.1 U/L high from non-high turnover. Appropriate cut-off values using the Ostase mass bALP (IDS iSYS) have been suggested and include <21 µg/L for discriminating low from non-low bone turnover and >31 µg/L for high from non-high bone turnover [43, 73].

#### FGF-23/phosphate axis and Wnt inhibitors

FGF-23 increases early in CKD to prevent hyperphosphataemia [74, 75]. As CKD progresses, the expression of klotho-FGFR1 in kidneys and parathyroid glands decreases suggesting a state of resistance to FGF-23’s actions in the regulation of PTH [76]. Both hyperphosphataemia and high FGF-23 concentrations, which are important components of the CKD-MBD syndrome, have been associated with increased mortality in dialysis patients, with the mortality risk being higher in patients with elevated FGF-23 concentrations compared to those with hyperphosphataemia (5–6-fold increase compared to 1.3–2) [77, 78]. Elevated FGF-23 levels have been independently associated with left ventricular hypertrophy and myocardial damage in CKD, which may explain, in part, the mortality risk [79]. Elevated FGF-23 concentrations have also been associated with increased risk of mortality in a prospective cohort study where only 16.4% of participants had eGFR of <60 mL/min/1.73 m^2^ [80]. Crucially, however, these associations do not support a causal effect, as a 2018 meta-analysis failed to show any exposure–response relationship between FGF-23 and cardiovascular or non-cardiovascular outcomes in populations with and without known CKD [81].

High serum phosphate contributes to low bone mass and high fracture risk, either directly [82] or indirectly through the modulation of PTH and FGF-23. FGF-23 regulates bone mineralisation as shown in experimental models [83]. In earlier studies, higher FGF-23 was associated with parameters of bone histomorphometry, including osteoid thickness and osteoid maturation time in children with CKD-5D with high turnover bone disease, suggesting that FGF-23 may reflect the skeletal mineralisation status in CKD-5D [84]. It inhibits tissue-non-specific ALP independently of Klotho [85], and is thought to contribute to bone loss by stimulating the Wnt inhibitor, DKK1 [86]. Indeed, studies show that Wnt inhibitors including DKK1 and sclerostin may link VC to bone loss in CKD [87]. Increased sclerostin expression has been demonstrated in VC and its secretion in calcified vessel may enter the circulation and contribute to low bone formation in CKD [88, 89]. These biomarkers may prove useful in better understanding CKD-MBD, but further studies are needed before they can be established in clinical practice.

## Risk factors for fractures in CKD

Clinical non-BMD risk factors for osteoporosis and fractures also apply to patients with CKD and should be evaluated in fracture risk assessment. These traditional clinical risk factors are shown in Table 1. They are useful for case finding and the use of the clinical risk factors with the addition of BMD provides an optimal assessment. The FRAX algorithm (www.shef.ac.uk/FRAX) integrates these risk factors and BMD and derives the 10-year probability of a major osteoporotic fracture or hip fracture.

**Table 1:** Clinical risk factors for fractures in CKD and osteoporosis

Osteoporosis-related traditional risk factors	Factors with a higher prevalence in CKD
Female gender	Secondary hyperparathyroidism
Age	Dialysis-related and uraemic factors
Early untreated menopause	Metabolic acidosis
Low body mass index (BMI)	Malnutrition
Smoking	Peripheral neuropathy/muscular weakness/poor balance
Alcohol > 3 units/day	Increased risk of sarcopenia and falls
Hypogonadism	Neurocognitive dysfunction
Glucocorticoid use	Diabetes mellitus
Secondary risk factors: inflammatory diseases, malabsorption, rheumatoid arthritis	Cardiovascular disease; vascular calcification

### Use of FRAX in CKD

There are limitations to the use of the FRAX tool in CKD as it does not capture many of the non-traditional CKD-specific risk factors (Table 1) and fracture risk may be underestimated in patients with CKD stage 4/5. Nevertheless, the use of FRAX in CKD has shown promising results in terms of its predictive value, with best results obtained with FRAX plus BMD at the femoral neck. In a study from the CaMos with a mean follow up of 4.8 years for incident major osteoporotic fracture, the 5-year observed major osteoporotic fracture risk was 5.3% in those with an eGFR of <60 mL/min and this was comparable to the FRAX score (6.4% with BMD; 8.2% without BMD) [10]. In a more recent study in a larger cohort (the Manitoba Bone Mineral Density Database), the usefulness of the FRAX score derived with and without BMD was assessed in relation to CKD stage 3 (*n* = 2154) and stage 4–5 (*n* = 590). Incident fractures (major osteoporotic fractures and hip fractures) were recorded over a 5-year period. FRAX was found to predict major osteoporotic fracture and hip fracture across all eGFR groups [90]. The interaction between the probability of a major osteoporotic fracture and eGFR was significant with stronger associations with FRAX in patients with CKD compared to those without (*P* < 0.001), although FRAX underestimated fracture risk in those with eGFR < 30 by 2.5% [71]. The study did not show any improvement in FRAX prediction across all eGFR categories when BMD was included [71]. To enhance fracture risk assessment using FRAX, several arithmetic adjustments have been proposed that can be applied to the conventional FRAX algorithm. These include, for example, glucocorticoids dose, lumbar spine BMD, HAL, falls history, recency of fracture, diabetes and CKD [91], and study results support the use of FRAX in non-dialysis CKD to identify patients who are at high risk of fracture. Additional studies are needed to assess its predictive ability in dialysis patients where derangements of calcium and phosphate homoeostasis and secondary hyperparathyroidism are more pronounced.

## Intervention for fracture reduction in CKD

### Correction of modifiable traditional risk factors

As with the general population, lifestyle measures to improve bone health also applies to patients with CKD. This should include advice targeted towards traditional risk factors such as increasing the level of physical activity, smoking cessation, reducing alcohol intake to ≤3 units/day, the risk of falls and optimising calcium/vitamin D intake. Falls risk assessment should form an integral part of any fracture reduction strategy [92].

Calcium and vitamin D supplements are often prescribed as adjuncts to osteoporosis treatment. Calcium supplementation is generally considered if dietary calcium is below 700 mg/day, and a calcium intake between 700 and 1200 mg is recommended [73]. In CKD, extra caution is advised because of impairment in calcium homoeostasis [93]. Formal calcium studies in adults with CKD found that maintaining an oral calcium intake between 800 and 1000 mg by diet or calcium containing phosphate binders leads to neutral balance and should not be exceeded, whereas calcium intakes of 1000 mg or more result in positive balance [94, 95]. In the general population, vitamin D supplementation alone has minimal effects on fractures and falls unless in severe deficiency, although calcium and vitamin D combined has been shown to have modest anti-fracture efficacy [96]. In the non-CKD population, although vitamin D (cholecalciferol) supplement of 800 IU daily is advised as maintenance dose in those who are at high risk of fracture risk [73], it is important that they are also calcium replete to maximise skeletal benefits. A similar recommendation might be made for patients with CKD stages 1–4 due to the positive effects of improved vitamin D status on other outcomes, even in the absence of fracture data. By contrast, the use of intermittent bolus doses of vitamin D (≥100,000 IU), convenient as it may seem, is not recommended in either population because of the associated increased falls and fracture risk [97, 98].

### Management of biochemical abnormalities of CKD-MBD

Treatment in patients with CKD should focus on reversing or reducing the extent of biochemical abnormalities associated with CKD-MBD, before considering specific treatment options for osteoporosis and fractures, as recommended by the 2017 KDIGO guidelines [3]. This includes the improvement of vitamin D deficiency, hyperphosphatemia and hyperparathyroidism. In patients with pre-dialysis CKD, low 25(OH)D (<15 ng/mL) has been associated with 1,25-dihydroxyvitamin D concentration of <16.7 pg/mL (ref range: 16.7–33.8) [99]. Data also suggest that administration of cholecalciferol or 25(OH)D in CKD-5D increases calcitriol concentration, suggesting that despite the thousand-fold difference in molar concentrations, substrate availability may still be important for extra-renal calcitriol synthesis [100, 101]. Dual supplementation with nutritional and active vitamin D (calcitriol or other vitamin D analogues) can be used to control worsening secondary hyperparathyroidism to keep serum PTH within the recommended cut-off values in CKD-5D. Treatment should be monitored to prevent hypercalcaemia and hyperphosphataemia, which can result in soft tissue and vascular calcification and the development of ABD [80]. These drugs can also be used with caution when there are continuing trends to increased PTH in CKD stages 4 and 5 [67, 102]. The use of one-alfa calcidol has been shown to improve bone histology in CKD 4 and has similar activity to calcitriol in CKD-5D [80]. Other vitamin D analogues such as paricalcitol have been shown to suppress PTH, although no significant difference in outcomes has been found between the newer and more established vitamin D compounds in CKD-5D or in pre-dialysis CKD [103, 104]. Despite the maintenance of PTH within recommended ranges being a major focus of CKD clinical management, there have been no randomised controlled trials to demonstrate whether the use of vitamin D analogues or receptor agonists reduces fractures. The calcimimetic drug cinacalcet has been reported to reduce secondary hyperparathyroidism similarly to the vitamin D compounds, although it is not recommended as first line therapy for secondary hyperparathyroidism [3]. The evaluation of cinacalcet therapy to lower cardiovascular events (EVOLVE) study did not show any significant reduction in mortality and cardiovascular events following intention to treat analysis, in dialysis patients. Similarly, cinacalcet did not reduce fracture rates in unadjusted intention to treat analyses, although when adjusted for differences in baseline characteristics and clinical events leading to discontinuation of cinacalcet, a significant reduction in fractures was reported [105]. Cinacalcet has been shown to improve BMD at the femoral neck in a small study of patients on haemodialysis [106].

Lowering elevated serum phosphate values towards the normal range by dietary means, dialysis or the use of phosphate binders can all be considered in patients with CKD. These interventions can reduce risks of hyperparathyroidism and may reduce vascular calcification risks [3]. A Cochrane review found that when compared to calcium-based binders, the use of sevelamer was associated with a lower risk of death (all causes) and hypercalcaemia in CKD-5D, which persisted when the analysis was restricted to studies at low risk of bias. However, the effects of phosphate binder use on vascular calcification, cardiovascular mortality, myocardial infarction, stroke and fracture risk remain unclear [107, 108].

Correcting CKD-MBD biochemical abnormalities and controlling PTH have not been shown to significantly improve fracture risk, except possibly with the use of cinacalcet. Pharmacological agents used for fracture prevention in people with osteoporosis should be considered in CKD patients who are at high risk of fracture. However, CKD-related biochemical derangements should first be corrected or improved, as this may improve responses, reduce complication rates and reduce risks for vascular calcification.

### Use of osteoporosis medications for fracture prevention in CKD

Treatment of osteoporosis in patients with CKD stages 1–3 is the same as that in patients without CKD, unless there is evidence of the laboratory abnormalities described above which should be addressed first [3]. The use of osteoporosis medications in the context of advanced CKD stages 4–5D is complex and challenging. First, most anti-fracture treatments are not recommended for patients with an eGFR < 30 mL/min/1.73 m^2^ because of reduced renal clearance, although most are dialysable. When given during dialysis as an intravenous infusion, 50% of alendronate is removed during the dialysis session, which is similar to its renal elimination in patients with normal renal function [109]. Bisphosphonates are also cleared by peritoneal dialysis [110]. However, if a bisphosphonate is given post-dialysis, there is a difference in retention due to the (often 2–3 days) interval before the next dialysis session. This may lead to higher binding of bisphosphonate to bone [111]. In this situation, reducing/halving the dose of bisphosphonate or frequency of administration may be required to allow for retention of a dose equivalent to that prescribed for patients with normal renal function, although clinical trial data are lacking. This has led to some therapeutic nihilism in bone clinics, since patients are often perceived as out of bounds in terms of our general armoury of osteoporosis medications, and improving the biochemical components of CKD-MBD is generally managed by nephrologists. This suggests that a multidisciplinary approach incorporating endocrine, rheumatology and renal expertise may be needed. A brief list of therapeutic agents available for fracture prevention in the general population and their GFR cut-offs is summarised in Table 2.

**Table 2:** Summary of available drugs for use in fracture prevention in osteoporosis

Drugs	Dosage	Approved GFR cut-off, mL/min	Predominant mode of action on bone
Bisphosphonates			
Alendronate	70 mg weekly oral	>35 mL/min	Anti-resorptive
Risedronate	35 mg weekly oral	>30 mL/min	Anti-resorptive
Ibandronate	150 mg monthly oral or 3 mg every 3 months iv	>30 mL/min	Anti-resorptive
Zoledronate	5 mg annually but may require less frequent dosing based on bone turnover markers	>35 mL/min	Anti-resorptive
Denosumab^a^	60 mg every 6 months subcutaneous	Any GFR	Anti-resorptive
Raloxifene	60 mg daily oral	Not endorsed for use in patients with severely impaired renal function	Anti-resorptive
Hormone replacement therapy (HRT) (male and female)/menopausal hormonal therapy (MHT)	Differing doses and preparations; oral, transdermal, continuous or sequential	Any GFR	Anti-resorptive
Teriparatide	20 µg daily subcutaneous daily for up to 2 years	>30 mL/min	Anabolic
Romosozumab	210 mg subcutaneously monthly for 12 months	Not known	Anabolic

^a^No dose adjustment is required in patients with renal impairment. Serum calcium should be monitored in patients with severe renal impairment or receiving dialysis

#### Anti-resorptive agents

Anti-resorptive agents inhibit osteoclast-driven bone resorption. The most potent classes of anti-resorptive drugs are nitrogen-containing bisphosphonates, which chemically interfere with intracellular osteoclast function through inhibiting protein prenylation [112], and denosumab, which blocks osteoclast activation through the critical RANK pathway by binding RANK ligand [113]. All bisphosphonates are excreted by the kidneys [114]. Concerns about the accumulation of bisphosphonates in CKD, with consequent oversuppression of bone turnover, have led to avoidance of their use in patients with an eGFR < 30 mL/min. However, post-hoc analyses of nine randomised controlled trials of risedronate in post-menopausal women with osteoporosis, categorized into three GFR groups based on the Cockcroft–Gault equation (which incorporates age), showed that all three groups had a significant increase in BMD and vertebral fractures reduction compared to placebo [115]. The GFR groups were mild (50 to <80 mL/min), moderate (30 to <50 mL/min) and severe (<30 mL/min). Risedronate did not adversely affect renal function, and adverse events were similar in the placebo and risedronate arms irrespective of renal function. Suppression of bone turnover was not different between the three groups and histomorphometric analysis from paired biopsies in a small group of participants did not reveal any mineralisation defects or features of ABD [90]. Similar data have also been observed in a post-hoc analysis of pooled clinical trial data of risedronate use in 852 participants with osteoporosis from Japan [116]. Post-hoc analysis of the fracture prevention trial (FIT) in post-menopausal women with osteoporosis revealed that alendronate increased BMD irrespective of renal function [117]. Women with GFR < 45 mL/min had an improvement in total hip BMD by 5.6% compared with 4.8% among women with normal to moderate renal dysfunction; GFR > 45 mL/min (*P* = 0.04). Alendronate treatment reduced the risk of clinical fractures across all renal impairment sub-groups (OR = 0.8; 95% CI: 0.70–0.9) compared to placebo, although none of the women had GFR < 30 mL/min [92]. There were no significant differences in adverse events or deterioration in renal function between groups. However, in a recent study of two cohorts with moderate and severe CKD (eGFR < 45 mL/min), bisphosphonate use was associated with a modest increased risk of CKD progression of 15%, although the number of subjects with CKD stage 5 was small (*n* = 45). No other safety concerns were reported in the study population including acute kidney injuries. These findings suggest that in the absence of laboratory abnormalities of CKD-MBD, bisphosphonates may be used with caution and appear to be effective in patients with renal impairment due to age-related decline and in patients with eGFR < 30 mL/min/1.73 m^2^ [118]. However, whether these findings can be extrapolated to patients with CKD stage 5 and those with renal impairment due to specific, intrinsic renal disease remains less clear. There have been a few small studies in patients with CKD-MBD, although results are inconclusive. An increase in lumbar spine BMD (*T* score) following alendronate for 18 months has been observed in a small study of patients with CKD 3–4, although change in BMD was a secondary outcome [119]. In a study of patients (*n* = 16) on dialysis, iv ibandronate (2 mg every 4 weeks) for 48 weeks increased BMD and reduced bone turnover. No significant change in PTH was observed [120]. A recent randomised controlled trial of intravenous alendronate (900 µg/4 weeks) for 1 year compared to denosumab in 48 patients on haemodialysis showed that in the alendronate group, lumbar spine BMD increased significantly from baseline to 6 months by 2.7% and to 12 months by 5.4%, with similar increases in the denosumab group of 2.8% and 5.6% at 6 and 12 months, respectively [121]. There was a significant reduction in bone turnover markers at 3 months (TRACP-5b: −11.9%, *P* < 0.05; PINP: −21.1%, *P* < 0.01; bALP: 9.9%, *P* < 0.05). PTH concentrations following alendronate did not differ from baseline [121]. Further trials are registered, though not yet recruiting, (ACTRN12620000321943) investigating the effect of risedronate on parameters associated with fracture risk such as BMD, HSA, TBS and the aortic calcification score in patients on dialysis. In studies of kidney transplant patients who are at risk of bone loss, particularly in the first year post-transplantation, bisphosphonate was shown to improve BMD at the lumbar spine and one study showed that iv ibandronate (3 mg every 3 months for 12 months) administered in the first year following transplantation prevented bone loss at the femoral neck and radius with modest effect [122]. However, fracture prevention following bisphosphonate in patients with CKD 3–5 and renal transplantation remains unclear as the studies did not have sufficient power to show any reduction in incident fractures. Furthermore, bone biopsy data in patients with CKD-MBD following bisphosphonates are not available.

In contrast to bisphosphonates, denosumab is not cleared by the kidneys or accumulated in bone tissue. There is therefore no theoretical risk of accumulation in CKD. Indeed, renal impairment does not significantly alter the pharmacokinetics of denosumab and no dose adjustments are required in CKD [123]. Denosumab does not appear to adversely affect renal function. A recent uncontrolled cohort study reported that in elderly people with normal renal function, 24 months treatment with denosumab was associated with an increase in cystatin C eGFR of 2.75 ± 1.2 mL/min/1.73 m^2^, which was also associated with a decrease in serum phosphate [124]. The decline in serum phosphate is unsurprising since calcium and phosphate are relocated to the skeleton upon reduction in activation frequency, with filling of the resorption space. However, in the absence of a control population or a plausible physiological mechanism, the effect of denosumab to improve renal function is speculative. The effect of denosumab on BMD in moderate CKD has been studied in a controlled setting. In a post-hoc analysis of the FREEDOM trial, the pivotal trial of denosumab in post-menopausal osteoporosis, women with CKD stage 3 (*n* = 2817) and CKD stage 4 (*n* = 73) using the Cockcroft–Gault equation, had a significant increase in BMD after treatment for 36 months, which did not differ by kidney function [125]. Vertebral fracture risk reduction was observed in CKD stage 3 (OR: 0.26–0.59) but not in those with CKD stage 4 as numbers were small. There were no differences in serum calcium and incidence of adverse events by CKD stage. However, these women did not have biochemical features of CKD-MBD including secondary hyperparathyroidism. A ‘real-life’ study in patients with CKD stage 4 showed that the response in BMD at the hip was significantly lower compared to patients with eGFR > 30 mL/min and this was negatively associated with PTH concentrations [126]. Several other studies have also shown an increased risk of hypocalcaemia following denosumab, particularly in patients with CKD-MBD and patients on dialysis. A single dose administration of denosumab in patients with various degrees of renal impairment ranging from mild to severe, led to hypocalcaemia in 15%, with a trend to increased hypocalcaemia as the severity of renal impairment increased. Hypocalcaemia was reported in 8, 23, 22 and 25% of patients with mild, moderate, severe and dialysis-dependent CKD [123]. In a 6-month study of 12 patients on dialysis, denosumab improved bone density significantly at the femoral neck and lumbar spine, although the most common adverse event was hypocalcaemia requiring increases in calcitriol, calcium supplementation and dialysate calcium [127]. PTH also increased initially but subsequently decreased following increases in calcitriol dosing. A 24-month trial of denosumab in dialysis patients reported a reduction in bone turnover markers. Serum calcium decreased by day 20 reaching a nadir at 2 months, needing close monitoring and rapid adjustment of calcium and vitamin D [128]. In kidney transplant recipients, denosumab was shown to increase BMD to a similar extent as other patient groups [129]. Although there was an increased incidence of cystitis and asymptomatic hypocalcaemia, the rate of graft rejection, opportunistic infections, urosepsis and pyelonephritis was similar to that of the untreated arm of the trial.

Early, regular and close monitoring is needed following denosumab, together with aggressive replacement of calcium and treatment with calcitriol or its analogues to prevent hypocalcaemia. Biochemical abnormalities of CKD-MBD should be reversed or reduced, prior to commencement of the drug to optimise skeletal response and prevent hypocalcaemia. These data demonstrate that denosumab can prevent BMD loss in patients with CKD 4, 5/5D, although the effect on fracture risk reduction remains uncertain. Clinical trials evaluating the effect of denosumab on bone and vascular metabolism in women with osteoporosis on haemodialysis (NCT02792413) are ongoing and the results should provide further information on its effects on BMD and fracture risk. Another important consideration in treatment planning is the ‘rebound’ bone loss and fractures which occur soon after denosumab is stopped, as reported in patients with osteoporosis, and which also applies, possibly with even greater impact, to the CKD population [130].

Raloxifene is a selective oestrogen receptor modulator and a less potent anti-resorptive agent compared to denosumab and bisphosphonates. It has not been shown to significantly reduce the risk of non-vertebral or hip fractures [131]. Post-hoc analysis of the MORE trial of raloxifene showed that treatment was associated with a greater increase in spine BMD and a reduction in vertebral fractures, irrespective of renal function [132]. Raloxifene had a greater effect on hip BMD among those with mild to moderate CKD, although data in CKD stage 4 are limited. Small, short-term studies of raloxifene in CKD and ESRD have demonstrated improvement in spine BMD [133], although evidence for a fracture benefit is low because of the high risk of study bias [105]. The European Medicines Agency does not endorse the use of raloxifene in patients with severe renal impairment and caution is warranted in moderate or mild renal impairment. The excess risk of venous thrombosis and the risk of fatal stroke observed in the RUTH trial is an additional concern in a population at elevated risk of atherosclerosis and cardiovascular events [134]. Menopausal hormone therapy or hormone replacement therapy (HRT) in women with normal renal function leads to reductions in vertebral and non-vertebral fractures by about 30% and this effect appears more marked in women younger than 60 years [135, 136]. Although concerns still exist about the increased risk of breast cancer with duration of use and risks of venous thromboembolism (VTE), the benefits of HRT may outweigh these risks in younger post-menopausal women [137]. The majority of women with CKD stages 5 and 5D are in the post-menopausal age range, but early menopause or amenorrhoea due to disruption in gonadotropin-releasing hormone secretion may occur in all CKD stages [138, 139]. While HRT should be considered in younger amenorrhoeic women with CKD or those who have undergone early menopause, the use of HRT in this group is less than in the general population. This may be related to concerns surrounding the risk of VTEs or clotting of vascular access in dialysis patients, although data are lacking. Longitudinal studies are thus needed to evaluate the efficacy and side-effects of HRT in women with CKD stages 4–5D. Beta-blockers have also been associated with improved BMD and reduced fracture risk, including in a large cohort from the Framingham Osteoporosis study. As many patients with CKD are on anti-hypertensive medications including beta-blockers, a clinical trial of beta-blockers with improvement in BMD and fracture risk as outcomes should be explored in the CKD population [140].

The use of potent anti-resorptive agents like denosumab and bisphosphonates has raised concerns, particularly in the CKD population, of promoting ABD, and increasing ABD-associated fractures and vascular calcification, although evidence for this is lacking. It can be argued that the net flow of calcium and phosphate in the CKD population, and in the general population aged 35 or over is not into the skeleton but out of the skeleton, hence lowering a high turnover state will not deliver higher calcium or phosphate to the vasculature and the effects of going from a low turnover state to a no turnover state will result in either no change in calcium and phosphate or a small decrease. The hypocalcaemia issue seen with denosumab in CKD is a clear illustration of the direction of flow. The anti-fracture efficacy of bisphosphonate (risedronate) is similar irrespective of bone turnover [141]. Whether this can be extrapolated to patients with CKD-MBD is unclear and trials of the use of anti-resorptive agents in patients with CKD and low bone turnover are needed. The use of bisphosphonates at lower dose or for shorter time periods (<3 years) could be an option in this setting although clinical trials are needed. Denosumab or alendronate have not been shown to increase vascular calcification in dialysis patients and CKD stages 3 and 4 [121].

#### Anabolic agents

Anabolic agents stimulate bone formation and are the treatment of choice in patients with osteoporosis at high risk of fracture. This includes teriparatide, which is a recombinant 1–34 N-terminal sequence of human PTH [142], and romosozumab, which is a monoclonal Ab against the Wnt signalling inhibitor sclerostin [143]. Their use is theoretically promising in CKD patients as they are not associated with low turnover or ABD.

Data from the fracture prevention trial show that teriparatide prevents vertebral and non-vertebral fragility fractures in post-menopausal osteoporosis [144]. Its use in CKD is controversial, particularly in patients who have secondary hyperparathyroidism where teriparatide could enhance loss of bone at cortical sites by increasing cortical porosity [145]. Post-hoc analysis of the fracture prevention trial in patients with mild or moderate renal impairment (GFR 50–79 and 30–49 mL/min/1.73 m^2^, respectively) showed that teriparatide increased P1NP, BMD at the lumbar spine and femoral neck and reduced vertebral and non-vertebral fracture risk, independently of renal function in the setting of normal PTH. These outcomes did not differ significantly from patients with normal renal function [146]. An increase in the incidence of hyperuricaemia was observed and was highest in those with moderate renal impairment. A post-marketing surveillance study in Japan of 1847 patients with osteoporosis at high risk of fracture included patients with osteoporosis and stage 4 (*n* = 30) or 5 (*n* = 3) CKD [147]. BMD increased in patients with CKD stage 4 as did P1NP at 3 months, although numbers tested were small (*n* = 6). No serious adverse drug reactions were recorded in patients with CKD stage 4 or 5, but hyperuricaemia and hypercalcaemia were noted [114]. A few small studies have looked at the use of teriparatide in patients (*n* = 7) with CKD-MBD in the context of ABD, where increases in lumbar spine and hip BMD were seen with no effects on vascular calcification [148]. Teriparatide (once weekly) also increased bone turnover markers as well as lumbar spine BMD in patients with CKD stage 5D with low BMD and hypoparathyroidism due to parathyroidectomy [149]. Further studies are needed to explore the use of teriparatide on fracture and cardiovascular risks, particularly in patients with CKD-MBD with low bone turnover before any definitive conclusions can be reached.

Studies show that romosozumab increases P1NP with a peak at 4 weeks and decreases thereafter. There is a decrease in CTX after the first week of romosozumab and CTX remains below baseline values during 12 months of treatment, suggesting uncoupling of bone remodelling in favour of net bone formation [110]. QCT measurement of bone structure showed that romosozumab increased cortical bone density compared to teriparatide [150]. In the large phase III trial, the fracture study in Post-menopausal Women with Osteoporosis (FRAME), women randomised to monthly subcutaneous injections of 210 mg of romosozumab for 12 months had a reduction in new vertebral fractures and clinical fractures by 73 and 36% compared to placebo [151]. In the second year, both groups were given denosumab, and vertebral fracture risk at 24 months was lower in participants who had been treated with romosozumab in the first 12 months [118]. Post-hoc analysis also showed a trend for reduction in non-vertebral fractures. Gains in BMD were significantly larger in women who had received romosozumab followed by denosumab. Mild transient reductions in serum calcium have been observed. For this reason, hypocalcaemia or vitamin D deficiency should be corrected prior to treatment and serum calcium should be monitored. A study comparing romosozumab and teriparatide (STRUCTURE) for 12 months demonstrated that gains in BMD and hip strength were significantly greater in the romosozumab group [152]. In the Active-Controlled Fracture Study in Post-menopausal Women with Osteoporosis at High Risk (ARCH), women randomised to romosozumab had significantly lower vertebral fracture risk, clinical fracture risk and non-vertebral fracture risk at 12 months compared to the alendronate group [153]. Vertebral fracture and clinical fracture risks remained lower at 24 months in the romosozumab to alendronate group compared to the alendronate-alendronate group. Post-hoc analysis of the FRAME study in women with mild (eGFR 60–89 mL/min) CKD stage 2, moderate (eGFR 30–59 mL/min) CKD stage 3 or severe (eGFR 15–29 mL/min) CKD stage 4 renal insufficiency showed that increases in BMD and reductions in new vertebral fractures were not affected by the level of renal impairment [154].

The incidence of adverse events did not differ by GFR sub-groups. However, in the ARCH study, an increase in serious cardiovascular events (myocardial infarction and stroke) was reported in patients treated with romosozumab compared to alendronate in the first 12 months (50 of 2040 patients on romosozumab [2.5%] versus 38 of 2014 patients on alendronate [1.9%]) [153]. Unfortunately, this will limit its use in patients with CKD-MBD as consideration should be given to cardiovascular risk based on prior cardiovascular disease, hypertension, hyperlipidaemia, diabetes mellitus, smoking and severe renal impairment.

Treatment choices are complex and difficult, requiring the treating physician to be familiar both with the principles of managing osteoporosis and CKD-MBD. Further, due to the paucity of trial data, the only alternative to therapeutic nihilism is to make informed choices based on what we understand about skeletal and mineral pathophysiology and about the modes of action of the therapeutics available to us. A proposed strategy to help guide treatment decisions in patients with CKD, based on available guidelines and the writers’ opinions, is shown in Tables 3 and 4. Among patients with stages 4–5D CKD who will also have biochemical abnormalities of CKD-MBD, the use of currently available drugs for the treatment of osteoporosis and fracture prevention would be ‘off-label’ either because the CKD patient group is not included or listed in the approved product information document and the use of the drug, particularly in the case of bisphosphonates, is outside the GFR limits set out in the authorised product information (the summary of the product characteristics). Off-label use of these drugs should take place only when the prescribing physician has considered all available options, and believes that the drug is being used for this condition in the best interest of the patient. This should also involve discussion and sharing of information with the patient and consent from the patient [155]. In this situation, the prescriber should also be able to defend his or her decision by submitting appropriate and relevant evidence to the respective institution’s drugs and therapeutic committee if required.

**Table 3:**
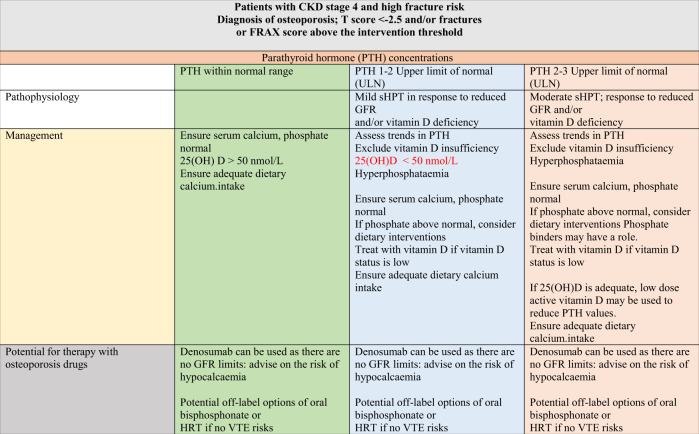
Proposed strategy to guide treatment decisions in patients with CKD stage 4 who are at high risk of fracture

**Table 4:**
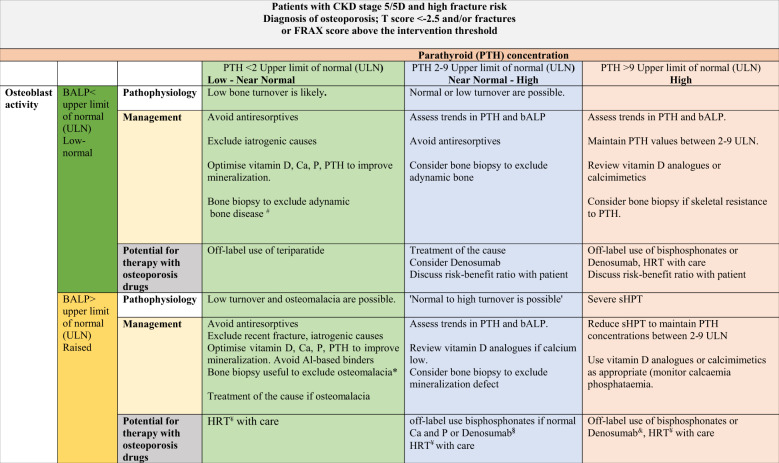
A proposed strategy to guide treatment decisions in patients with CKD stage 5/5D who are at high risk of fracture

We have developed this algorithm as a function of PTH concentration and bALP. The upper limit of PTH concentration in CKD stage 5/5D is ninefold the upper limit of normal (ULN) in accordance with the KDIGO guidelines which is used as threshold, but it is important to review trends and initiate treatment if there are changes in PTH in either direction before levels progress outside the 2–9-fold range. BALP is used as an additional marker of bone turnover. We use bALP value of lower than the upper limit of normal (ULN) as the cut-off value to differentiate between low versus non-low bone turnover. Similar cut-off values (<ULN) can be used if total ALP is used as an initial screening test. Both tests may be used as surrogates for bone biopsies in some patients before institution of anti-fracture agents. However, the biomarkers may not identify patients with mineralisation defects, in which case a bone biopsy may be needed prior to institution of anti-resorptive treatment

*PTH* parathyroid hormone, *bALP* bone-specific alkaline phosphatase, *sHPT* secondary hyperparathyroidism, *ULN* upper limit of normal, *Ca* serum calcium, *P* serum phosphate, *Al* aluminium

^a^In patients with CKD stage 5D, if PTH is <2 ULN and bALP < ULN, PTH can be increased by reducing active vitamin D analogues, dialysate Ca (see KDIGO guidelines regarding this), supplemental calcium as phosphate binders or cinacalcet

^b^Ensure that serum 25(OH)D values are sufficient, correct acidosis and hypophosphataemia if present, exclude aluminium exposure. Bone biopsy is the best approach

^c^HRT can be used in some patients if there is no predisposition to VTEs

^d^Off-label use of bisphosphonate can be discussed with the patient, as can denosumab, highlighting the risk of hypocalcaemia and rebound bone loss with denosumab, review risk–benefit ratio with patient

^e^Improve serum phosphate towards the normal range. If denosumab is used, careful supervision is needed to avoid severe hypocalcaemia, review risk–benefit ratio with patient. Management/follow up should involve a multidisciplinary team

To take into account differences in commercial PTH assays, different units and reference ranges, we have used fold differences from the assay ULN. Cut-off values for PTH in CKD stages 5 and 5D are 2–9-fold the assay ULN as per the KDIGO guidelines. However, it is important to view the trends in PTH, as progressive changes in PTH within this ‘acceptable’ range should warrant initiation or change of treatment to minimise the risk of PTH values falling outside this range. PTH values may progressively rise or increase acutely when potent anti-resorptive agents like denosumab are used. If PTH trends continue to increase, treatment with an active vitamin D, with calcium supplements or cinacalcet can be considered, depending on prevailing serum calcium and phosphate values.

The use of osteoporosis drugs in patients with low BMD, bone loss and fragility fractures plus CKD-MBD should be considered together with factors such as age and menopausal status (influencing the potential use of HRT), and whether other aspects of CKD-MBD can be improved. Bone biopsy may be useful in cases where low bone turnover or mineralisation disorders are suspected. Risks and benefits of treatment strategies should be explained to patients. If HRT is to be considered in the younger post-menopausal women with CKD 4–5D or women who have an early menopause, the risks of breast cancer and not least VTE should be discussed. It is unclear if there is a place for testosterone replacement in men with CKD and more research is needed—small studies suggest benefits in terms of erythropoetin and haemoglobin levels but there is no trial data on skeletal outcomes in men with ESRD [156]. In the case of bisphosphonates use off-label, discussion should involve the risk of prolonged suppression of bone remodelling due to renal retention of the drug, which may require dosage adjustment (halving of the dose) or reducing dosing frequency and limiting duration of treatment to 2–3 years. As for denosumab, although no change in dosing protocol is needed, the risk of hypocalcaemia is high in patients with more severe CKD and rebound bone loss and fractures on treatment cessation need to be considered, as do possible means to transition off treatment if necessary. Denosumab in this scenario may be better targeted to older patients with severe CKD, although hypocalcaemia is a significant concern. In the case of the anabolic agent romosozumab, CKD patients should be made aware of the increased risks of serious cardiovascular events in trials of PMO and this may well speak against use in a large proportion of CKD patients pending more safety data in this group. Off-label prescription of teriparatide may be an option in those patients with low to normal bone turnover and/or serum PTH values that are appropriate to or lower than expected for their stage of CKD, with BMD *T* scores < −3 and multiple fractures, particularly if they have sustained a fracture while on denosumab or bisphosphonate, although this remains to be tested. For both romosozumab and teriparatide, follow up treatment with an anti-resorptive drug is advisable, once the treatment course has been completed, and this should be part of the treatment plan. Does this mean that we should not consider 2 years of teriparatide in a CKD patient who does not tolerate anti-resorptives? First, providing the patient with a 2-year fracture free period despite chronic disease may be in the best interest of the patient even if bone mass cannot be maintained once teriparatide is stopped. Second, with underlying CKD there is the possibility that patients will return not to accelerated bone loss but to their pre-treatment state of low bone turnover, and maintain the effect of teriparatide without requiring an anti-resorptive agent. Further research is needed to answer these questions. Table 5 shows potential directions for future research.

**Table 5:** Suggested directions for potential future research in patients with CKD 4–5D who are at high risk of fracture

	Osteoporosis drug	Research question	Key outcomes	Secondary outcomes
CKD stage 4	Bisphosphonates Denosumab	Is the efficacy of bisphosphonate therapy influenced by maintenance of serum PTH, 25(OH)vitamin D and phosphate to target ranges?	Fracture incidence Percentage change in BMD DXA derived TBS, HSA Measures of bone quality (HR-pQCT, bone biopsies)	Laboratory parameters: renal function PTH, calcium/phosphate FGF-23 Bone turnover markers (bALP)
CKD stage 4	Bisphosphonates	Safety and efficacy of limiting treatment duration (2 years), halving the dose or reducing frequency of dosing interval?	Adverse events, Fracture incidence Percentage change in BMD DXA derived TBS, HSA Measures of bone quality (HR-pQCT, bone biopsies)	Laboratory parameters: renal function PTH, calcium/phosphate FGF-23 Bone turnover markers (bALP)
CKD stage 4	Anabolic agents: Teriparatide Abaloparatide Romosozumab	Safety and efficacy of anabolic agents following control of CKD-MBD?	Adverse events, Fracture incidence Percentage change in BMD DXA derived TBS, HSA Measures of bone quality (HR-pQCT, bone biopsies)	Laboratory parameters: renal function PTH, calcium/phosphate FGF-23 Bone turnover markers (bALP)
CKD stage 5/5D	Bisphosphonates	Safety and efficacy of bisphosphonates in low bone turnover? What dose, length of treatment, dosing interval? Percent clearance by the dialysis membrane?	Adverse events Fracture incidence Percentage change in BMD DXA derived TBS, HSA Measures of bone quality (HR-pQCT, bone biopsies) Progression of vascular calcification	Laboratory parameters: PTH, calcium/phosphate FGF-23 Bone turnover markers (bALP)
CKD stage 5/5D	Denosumab	Is the efficacy of denosumab therapy influenced by maintenance of serum PTH, 25(OH)vitamin D and phosphate to target ranges?	Adverse events Fracture incidence Percentage change in BMD DXA derived TBS, HSA Measures of bone quality (HR-pQCT, bone biopsies) Progression of vascular calcification	Laboratory parameters: PTH, calcium/phosphate FGF-23 Bone turnover markers (bALP)
CKD stage 5/5D	Anabolic agents: Teriparatide Abaloparatide Romosozumab	Safety and efficacy following control of CKD-MBD? And in low bone turnover?	Adverse events Fracture incidence Percentage Change in BMD DXA derived TBS, HSA Measures of bone quality (HR-pQCT, bone biopsies) Progression of vascular calcification	Laboratory parameters: PTH, calcium/phosphate FGF-23 Bone turnover markers (bALP)
CKD 4–5D	Sequential treatment denosumab followed by bisphosphonate teriparatide followed by bisphosphonate or denosumab	Safety and efficacy of sequential treatment in patients with CKD-MBD?	Adverse events Fracture incidence Percentage change in BMD DXA derived TBS, HSA Measures of bone quality (HR-pQCT, bone biopsies) Progression of vascular calcification	Laboratory parameters: TH, calcium/phosphate FGF-23 Bone turnover markers (bALP)

## Conclusion

Fracture risk and mortality due to fragility fractures, particularly, hip fractures, are increased in patients with CKD and ESRD. At first, the apparent increasing prevalence of low turnover CKD-MBD in today’s CKD-MBD spectrum would seem to argue against the use of anti-resorptive agents, but recent guidelines suggest that some concerns may be misplaced, and that more patients with osteoporosis and CKD may benefit from active treatment. This patient population combines classic risk factors associated with age-related osteoporosis and falls, together with those specific to CKD-MBD. Evaluation of fracture risk is recommended in CKD with measurement of BMD and the use of the FRAX assessment tool. Management should always include reversing or reducing the metabolic abnormalities. Although post-hoc analysis of data from pivotal trials of some current osteoporosis medications including anti-resorptive and anabolic agents suggests positive effects on fracture risk and BMD in CKD, there remain uncertainties about their use in patients with CKD-MBD. However, new guidelines are more proactive and the time for bone clinics to actively participate in the management of bone health for patients with CKD, with or without concomitant osteoporosis, has arrived. Management strategies for fracture prevention in patients with CKD-MBD will continue to require a multidisciplinary patient-centred approach. The risks and benefits of treatment initiation with osteoporosis agents need to be discussed with the individual patient and careful monitoring is required.
